# Predictive factors of recurrence for laparoscopic repair of primary and incisional ventral hernias with single mesh from a multicenter study

**DOI:** 10.1038/s41598-022-08024-3

**Published:** 2022-03-10

**Authors:** Micaela Piccoli, Francesca Pecchini, Gaetano Vetrone, Romano Linguerri, Giuliano Sarro, Umberto Rivolta, Amedeo Elio, Gianluca Piccirillo, Giuseppe Faillace, Emilia Masci, Davide Guglielminetti, Chiara Santorelli, Giorgio Soliani, Margherita Koleva Radica, Vincenzo Trapani, Domenico Marchi, Johanna Chester, Luca Leonardi, Silvia Neri

**Affiliations:** 1 Department of General Surgery, Emergency and New Technologies, Baggiovara General Hospital, AOU Modena, Modena, Italy; 2 Department of Surgery, Santa Maria Della Scaletta Hospital, Imola, Italy; 3 Department of General Surgery, Istituto Clinico di alta Specialità San Gaudenzio, Novara, Italy; 4 Department of General Surgery, ULSS Scaligera, San Bonifacio Hospital, Verona, Italy; 5 Department of Surgical Sciences, Unit of General Surgery, E. Bassini Hospital, Cinisello Balsamo, Milan, Italy; 6 Department of General Surgery and Laparoscopy, Malatesta Novello Clinic, Cesena, Italy; 7 Department of Surgery, Surgical Unnit 2, Azienda Ospedaliero Universitaria of Ferrara, Ferrara, Italy; 8 Department of Colo-Rectal Surgery, Policlinico Campus Bio-Medico, Rome, Italy; 9 Division of Dermatology, Department of Surgical, Medical, Dental and Morphological Sciences with Interest Transplant, Oncological and Regenerative Medicine, University of Modena and Reggio Emilia, Modena, Italy; 10 Department of General Surgery, Sassuolo and Area Sud Hospital, Modena, Italy

**Keywords:** Reconstruction, Quality of life

## Abstract

Laparoscopic ventral hernia repair (LVHR) is a widely practiced treatment for primary (PH) and incisional (IH) hernias, with acceptable outcomes. Prevention of recurrence is crucial and still highly debated. Purpose of this study was to evaluate predictive factors of recurrence following LVHR with intraperitoneal onlay mesh with a single type of mesh for both PH and IH. A retrospective, multicentre study of data collected from patients who underwent LVHR for PH and IH with an intraperitoneal monofilament polypropylene mesh from January 2014 to December 2018 at 8 referral centers was conducted, and statistical analysis for risk factors of recurrence and post-operative outcomes was performed. A total of 1018 patients were collected, with 665 cases of IH (65.3%) and 353 of PH (34.7%). IH patients were older (p < 0.001), less frequently obese (p = 0.031), at higher ASA class (p < 0.001) and presented more frequently with large, swiss cheese type and border site defects (p < 0.001), compared to PH patients. Operative time and hospital stay were longer for IH (p < 0.001), but intraoperative and early post-operative complications and reinterventions were comparable. IH group presented at major risk of recurrence than PH (6.7% vs 0.9%, p < 0.001) and application of absorbable tacks resulted a significative predictive factor for recurrence increasing the risk by 2.94 (95% CI 1.18–7.31). LVHR with a light-weight polypropylene mesh has low intra- and post-operative complications and is appropriate for both IH and PH. Non absorbable tacks and mixed fixation system seem to be preferable to absorbable tacks alone.

## Introduction

Abdominal wall hernias are defined as either congenital or primary defect of the abdominal fascia (primary hernia, PH) or acquired defect following abdominal surgery (incisional hernia, IH), with respective estimated incidences of up to 25%^1^ and 10–30%^2–4^. Ventral hernia repair has therefore become routine for general surgeons around the world despite the fact that management can be extremely challenging, especially in cases of complex incisional defects. Further, optimal surgical approaches of open or mini-invasive techniques are still under debate^5,6^.

Since its first introduction by Le Blanc et al. in 1992^7^, laparoscopic ventral hernia repair (LVHR) with intra-peritoneal onlay mesh (IPOM), has rapidly been affirmed as a safe alternative to open ventral hernia repair, and has the advantage of being highly reproducible and standardized^1,8,9^. LVHR is associated with lower morbidity rates^8,10^, less surgical site infections (SSI)^8–10,12^ and shorter hospital stay^8–11^, with similar recurrence rates^6,8,12^. In laparoscopic hernia surgery, patient selection, defect characteristics and the choice of mesh and fixation devices seem to influence surgical success and hernia recurrence risk. However, most reports of LVHR available in literature include heterogeneous patient cohorts with widely different hernia characteristics, treated with different surgical approaches utilizing several types of mesh and fixation devices^3,13^. Outcomes are often presented globally, despite evidence that PH and IH are two distinct pathological entities^4^. The impact of surgical approaches is therefore difficult to estimate and a standardized approach is advocated^6,14,15^.

The aim of this study is to retrospectively evaluate a multicentre Italian series of patients treated with a standardized LVHR technique and a single type of prosthetic mesh, according to pathological subgroups of PH or IH, with at least 12 months follow-up. The primary outcome is to analyze recurrence rates and identify specific risk factors for IH and PH. Secondary endpoints include the analysis of short and mid-term outcomes of laparoscopic IPOM technique for IH and PH. This study reports one of the largest series of laparoscopic IPOM approach with the application of a single mesh type, and an assessment of barriers to technical success.

## Methods

### Study design

A retrospective, observational, multicenter study of patients with PH or IH treated in 8 high volume Italian collaborating centers (highly specialized in laparoscopic repair of abdominal wall defects) with laparoscopic IPOM technique and the application of a single prosthetic mesh (Ventralight Echo PS™ (Bard, Davol Inc., Warwick, RI, UK), between January 2012 and December 2018 was performed. Inclusion criteria specified a minimum of 4 cm mesh overlap and 12 months follow-up. Demographic and clinical baseline, hernia, surgical, postoperative and follow-up characteristics and outcomes for selected patients were collected in a dedicated database. Study protocol was approved by the Ethics Committee of Baggiovara General Hospital—Modena and other centres involved, and conducted according to the principles of Helsinki, concerning human and animal rights. An informed consent was obtained from all participants and/or their legal guardians. All experiments were performed in accordance with relevant guidelines and regulations. Data was inserted into the database by the collaborating centers utilizing medical records.

### Patient inclusion

Included centers selected patients based on an intention to treat with laparoscopic repair. During the study period, mesh choice was often based on the surgeons’ choice material and in-house availability.

Baseline demographics included age, gender, body mass index (BMI), the American Society of Anesthesiologist (ASA) class, chronic obstructive pulmonary disease, cardiac comorbidities, diabetes mellitus, hypertension, cirrhosis, smoking status, oral anticoagulant and steroid therapy assumption and history of radiotherapy. Cardiac comorbidities were defined as history of congestive heart failure, angina or myocardial infarction.

Collected baseline hernia characteristics included the type of hernia: primary or incisional (primary or recurrent incisional). Any recurrent primary hernia was classified as an incisional hernia. Other hernia data included hernia size, location and swiss cheese type. Hernia size was grouped according to the European Hernia Society (EHS) classification^16^ as W1 (< 4 cm), W2 (≥ 4–10 cm) or W3 (≥ 10 cm) and hernia location was grouped as medial (subxiphoidal (M1), epigastric (M2), umbilical (M3), infraumbilical (M4), suprapubic (M5)), lateral (subcostal (L1), flank (L2), iliac (L3), lumbar (L4)), or combined. Border hernias were defined as subxiphoidal (M1), suprapubic (M5), subcostal (L1) and lumbar (L4)^17^.

Collected intraoperative surgical data included elective or emergent surgical repair (strangulated hernia requiring intervention within 6 h from admission), operative time, fixation system (absorbable, permanent or mixed), any concurrent interventions, intraoperative complications (bowel perforation/injury, major bleeding) and the need for conversion to open repair.

Post-operative outcomes ($\le$ 30 days from intervention) included post-operative complications, reinterventions, pain assessment, and duration of hospital stay. Post-operative complication assessment included abdominal wall hematoma, hemoperitoneum, peritonitis, prosthesis infection due to abdominal contamination, bowel obstruction, medical complications such as pleural effusion, pneumonia and acute renal failure. Post-operative pain was measured by Numerical Rating Scale (NRS) from 0 (no pain) to 10 (severe pain). Pain outcomes were grouped according to the following categories: none (0), mild (1–3), moderate (4–6), severe (7–10)^18^.

Late complications (> 30 days from intervention) included possible persistent and/or symptomatic seroma, adhesion occlusive bowel syndrome, prosthetic infection, chronic pain, bulging and hernia recurrence. Persistent/symptomatic seroma was defined when enduring > 6–8 weeks after surgery and requiring needle aspiration^17^; chronic pain was considered persisting pain for 6–8 weeks post-surgery^17^; bulging was defined as swelling in the area of previous laparoscopic repair due to protrusion through the hernia opening into the hernia sac^19^. Hernia recurrence was defined as any abdominal wall gap with or without bulge in the area of postoperative scar perceptible or palpable by clinical examinations or imaging, as proposed by Korenkov et al.^20^.

### Preoperative assessment

Pre-operative management included clinical evaluation and imaging diagnosis (ultrasound, computed tomography (CT) or magnetic resonance imaging (MRI)-scan) according to the surgeons’ choice. Prior to surgery, informed, written consent was obtained by all patients. Short-term single-dose antibiotic-prophylaxis with a 2nd generation cephalosporin and antithrombotic therapy (low-weight molecular heparin) were routinely administered.

### Standardized LVHR surgical technique

All interventions were performed by surgeons with experience in laparoscopic IPOM procedures for abdominal wall surgery. Procedures were performed under general anaesthesia, following a standardized technique for the main steps of the laparoscopic procedure. In most cases, three ports were used and positioned on the left side of the abdomen; if necessary, for example for large defects, one additional port was placed on the right side. A 30° laparoscopic camera was used. The first step was the exposition of the abdominal wall defect; adhesiolysis was performed when required and as recommended a ‘cold dissection’ was applied to prevent accidental enterotomies, using electrified instruments only at a safe distance from the viscera^16^. In case of leakage from the small bowel, a laparoscopic suture of the lesion was performed and prosthetic repair was completed. Accurate visualization of the entire laparotomic incision in case of incisional hernias, was advocated by all operators to exclude the presence of multiple defects. Isolation with dissection and reduction of hernia sac was conducted and all hernia defects were then measured in longitudinal and transverse directions and an adequate size mesh was selected. The selected mesh (VentralightTM ST mesh, Bard, Davol Inc., Warwick, RI, UK) is a light-weight (51 g/m^2^) monofilament microporous polypropylene mesh (pores vary between 300 and 1000 micron) with a 30 days-resorbable hydrogel barrier to minimize tissue attachment to visceral side of the mesh, which is activated when in contact with a saline solution. The mesh was introduced through a 12 mm port, oriented toward the defect and then applied with the positioning system (Echo PS™ Positioning System Bard, Davol Inc., Warwick, RI, UK) constituted by a pre-attached, low-profile balloon to help facilitate deployment, centering and correct placement of mesh. The mesh was placed in the intraperitoneal in an underlay position with the side of the hydrogel barrier towards the abdominal viscera, and then secured to the anterior abdominal wall with an overlap of at least 4 cm in all directions.

Type of fixation device (absorbable and non-absorbable tacks) varied at each collaborating center. Tacks were chosen according to surgeons’ preferences and registered as absorbable tacks only, non-absorbable or mixed fixation technique (ratio 1:1). The ‘double crown’ technique was routinely ensured. Mesh fixation was performed under a tension-free placement with the reduction of pneumoperitoneum to 8–10 mmHg. Closure of hernia defect, before the mesh placement, was not routinely performed. Hemostasis was achieved before removing the trocars and no abdominal or subcutaneous drains were placed. Nasogastric tube and bladder catheter were removed at the end of the surgical intervention.

### Post-operative management and follow-up evaluation

Each center managed post-operative analgesic treatment with on-demand oral and intravenous drugs. Post-operative wound and ventral compression were applied in the post-operative period. Oral analgesic therapy and patient mobilization were performed as fast as possible, and all patients were invited to continue to wear abdominal compressive dressing for at least 30 days after surgery. All patients were visited within 30 days from intervention. Throughout the follow-up, patients were either visited with clinical examination or received random phone contact from the operative centers. In case of suspect/certain hernia recurrence or surgical related complications, clinical evaluation and imaging were performed. All cases of hernia recurrence reported in this cohort were defined upon clinical examination and/or imaging (ultrasonography, CT scan or MRI). Reinterventions were scheduled and performed following an interdisciplinary discussion with either laparoscopic or open repair techniques. A final phone interview of enrolled patients was performed at study closure, July 2020. Data collected during the follow-up included any incidence of late complications or any eventual patient death. Mortality was defined according to death registration $\le$ 30 days (immediate/procedure related) or > 30 days (late) from surgery. Loss to follow-up was defined as patients unable to be contacted > 30 days from initial intervention: registration of the presence or absence of recurrence was assigned to the last available clinical visit or phone interview.

### Statistical analysis

All data were collected in an electronical database and processed by STATA program version 14 (StataCorp LP 4905 Lakeway Drive College Station, Texas 77845 USA). Numerical data were expressed as mean and standard deviation or median and range, as appropriate. Categorical data were expressed as frequency and percentage. Variables were assessed according to hernia type, IH or PH. Recurrent cases were compared with all non-recurrent cases. Chi-square test (Fisher’s exact test) was used to examine the relationship between categorical variables, statistical differences for continuous variable for groups were examined using Student’s t test. The Tukey–Kramer pairwise comparisons was applied for variable fixation studentized range with significance according to the calculated critical value. Survival analysis was performed using the Kaplan–Meier method and comparisons between recurrence was assessed with a log-rank test. Multivariate analysis was performed for incisional hernia only, as there were too few recurrent cases in primary hernia subgroup to justify analysis, and it was made according to the Cox-regression hazard model (including significant factors of from univariate analysis) expressed as Hazard ratio (HR) with it 95% confidence interval (CI). A p-value < 0.05 was considered significant.

## Results

### Overall patient cohort

A cohort of 1018 patients matched our inclusion criteria and were enrolled into the current study. Most interventions (60%) were performed between 2017–2018 and most (70.3%) were recruited from three collaborating institutions (22.5%, 34.2% and 13.6% respectively). The remaining collaborating centers reported an equal distribution of cases (range 5–7%).

Baseline demographic, clinical and hernia characteristics, intraoperative and post-operative outcomes for (i) all patients, (ii) cases of recurrence, IH and PH types (iii) are reported in Tables 1, 2 and 3.

**Table 1:** Patient baseline demographic, clinical and hernia characteristics for all patients (n = 1018), recurrences* (n = 47) with significative risk factors (p value), and comparison between IH (n = 665) and PH (n = 353).

	All patients		Recurrence*		Hernia type		
	n = 1018		n = 47	P value	IH (n = 665, 65%)	PH (n = 353, 35%)	P value
Age (years, median [Q1–Q3])	62 (51–71)		64 (50–72)	n.s	65 (54–73)	55 (44–67)	< 0.001
Age years, mean ± SD (range)	60.4 ± 13.6 (20–92)		61.5 ± 13.7 (37–90)	n.s	63.1 ± 12.6 (28–92)	55.4 ± 14.0 (20–83)	< 0.001
**Sex**							
Male	506 (49.7)		24 (51.1)	n.s	310 (46.6)	196 (55.5)	0.007
Female	512 (50.3)		23 (48.9)		355 (53.4)	157 (44.5)	
**BMI (mean kg/m**^**2**^**)**							
Normal < 30	622 (61.1)		28 (59.6)	n.s	421 (63.3)	201 (56.9)	0.031
Obesity ≥ 30	363 (35.7)		19 (40.4)		221 (33.2)	142 (40.2)	
BMI mean [± SD]	28.5 ± 5.5 (17–69)		28.5 ± 5.5 (20–43.5)	n.s	28.2 ± 5.5 (17–69)	29 ± 5.4 (18–49)	0.030
**ASA class**							
I	186 (18.3)		8 (17.0)	n.s	96 (14.4)	90 (25.5)	< 0.001
II	609 (59.8)		29 (61.7)		409 (61.5)	200 (56.7)	
III	216 (21.2)		8 (17.0)		156 (23.5)	60 (17.0)	
IV	3 (0.3)		1 (2.1)		2 (0.3)	1 (0.3)	
**Clinical comorbidities**							
COPD	47 (4.6)		1 (2.1)	n.s	31 (4.7)	16 (4.5)	n.s
Cardiac comorbidities	86 (8.4)		3 (6.4)	n.s	62 (9.3)	24 (6.8)	n.s
Diabetes mellitus	119 (11.7)		3 (6.4)	n.s	81 (12.2)	38 (10.8)	n.s
Hypertension	430 (42.2)		22 (46.8)	n.s	290 (43.6)	140 (39.7)	n.s
Current smoker	246 (24.2)		10 (21.3)	n.s	130 (19.5)	116 (32.9)	< 0.001
Oral anticoagulant	44 (4.3)		0 (0.0)	n.s	30 (4.5)	14 (4.0)	n.s
Steroid therapy	14 (1.4)		0 (0.0)	n.s	12 (1.8)	2 (0.6)	n.s
Radiotherapy	11 (1.1)		1 (2.1)	n.s	9 (1.4)	2 (0.6)	n.s
Chirrosis	7 (0.7)		1 (2.1)	n.s	6 (0.9)	1 (0.3)	n.s
**Hernia characteristics**							
PH	353 (34.7)		3 (6.4)	< 0.001	0 (0.0)	353 (100.0)	–
Primary IH	560 (55.0)		33 (70.2)		560 (84.2)	0 (0.0)	
Recurrent IH	105 (10.3)		11 (23.4)		105 (15.8)	0 (0.0)	
Swiss cheese	178 (17.5)		6 (12.8)	n.s	169 (25.4)	9 (2.5)	< 0.001
Adhesion syndrome	89 (8.7)		7 (14.9)	n.s	77 (11.6)	12 (3.4)	< 0.001
**Hernia defect size**							
W1 (< 4 cm)	506 (49.7)		16 (34.0)	0.073	192 (28.9)	314 (89.0)	< 0.001
W2 (≥ 4 to < 10 cm)	384 (37.7)		22 (46.8)		348 (52.3)	36 (10.2)	
W3 (≥ 10 cm)	128 (12.6)		9 (19.1)		125 (18.8)	3 (0.8)	
**Hernia localization**							
Medial	913 (89.7)		38 (80.9)	0.082	573 (86.2)	340 (96.3)	< 0.001
Lateral	84 (8.3)		8 (17.0)		81 (12.2)	3 (0.8)	
Combined	21 (2.1)		1 (2.1)		11 (1.7)	10 (2.8)	
Border hernia	47 (4.6)		5 (10.6)	0.044	46 (6.9)	1 (0.3)	< 0.001

*Comparison with all patients without recurrence (n = 971).

Data presented were n (%), unless specified. *Q1*; first quartile, *Q3*; third quartiles; *BMI*, body mass index; *ASA*, American Society of Anesthesiologists; *COPD*, chronic obstructive pulmonary disease; *n.s.*, not significative.

**Table 2:** Intraoperative details for all patients (n = 1018), recurrences* (n = 47) with significative risk factors (p value), and comparison between IH (n = 665) and PH (n = 353).

	All patients	Recurrence*		Hernia Type		
	n = 1018	n = 47	P value	IH (n = 66,565%)	PH (n = 353, 35%)	P value
**Intervention type**						
Elective	959 (95.0)	46 (97.9)	n.s	634 (96.2)	325 (92.9)	0.025
Emergent surgical repair	58 (5.7)	1 (2.1)		30 (4.6)	28 (8.0)	
**Operative time**						
(Median, IQR)	70 (60–100)	75 (60–120)		80 (60–120)	60 (50–75)	< 0.001
Mean ± SD (range)	80.9 ± 36.7 (19–300)	92.2 ± 44.0 (20–195)	0.032	89.2 ± 39.7 (19–300)	65.5 ± 23.7 (20–175)	< 0.001
**Fixation system**						
Absorbable	480 (47.2)	30 (63.8)	0.010	382 (58.0)	98 (28.0)	< 0.001
Permanent	385 (37.8)	8 (17.2)		177 (26.9)	208 (59.4)	
Mixed	153 (15.0)	9 (19.1)		106 (16.1)	47 (13.4)	
**Mesh overlap**						
Mean cm ± SD (range)	5.0 ± 0.8 (4–11)	5.3 ± 1.0 (4–9)	0.03	5.1 ± 0.8 (4–11)	4.8 ± 0.8 (4–10)	< 0.001
Closure of hernia defect	53 (5.3)	5 (10.6)	0.089	43 (6.5)	10 (2.9)	0.012
All concurrent laparoscopic procedures°	116 (11.5)	10 (21.3)	0.029	99 (15.0)	17 (4.9)	< 0.001
**Concurrent laparoscopic procedures**						
Adhesiolysis	89 (8.7)	7 (14.9)	n.s	77 (11.6)	12 (3.4)	< 0.001
Cholecystectomy	12 (1.2)	1 (2.1)	n.s	11 (1.7)	1 (0.3)	0.044
Inguinal hernia repair	10 (1)	2 (4.3)	0.046	9 (1.4)	1 (0.3)	n.s
Cholecystectomy + Inguinal hernia repair	2 (0.2)	0 (0.0)	n.s	2 (0.3)	0 (0.0)	n.s
Ovarian cyst asportation	1 (0.1)	0 (0.0)	n.s	1 (0.2)	0 (0.0)	n.s
Adrenalectomy	1 (0.1)	0 (0.0)	n.s	1 (0.2)	0 (0.0)	n.s
Appendectomy	1 (0.1)	0 (0.0)	n.s	0 (0.0)	1 (0.3)	n.s
All intraoperative complications	11 (1.1)	0 (0.0)	n.s	8 (1.2)	3 (0.9)	n.s
Bowel Perforation/injury	8 (0.8)	0 (0.0)	n.s	6 (0.9)	2 (0.6)	n.s
Major bleeding	3 (0.3)	0 (0.0)	n.s	2 (0.3)	1 (0.3)	n.s
Conversion to open repair	2 (0.2)	0 (0.0)	n.s	2 (0.3)	0 (0.0)	n.s

Data presented were n (%), unless specified. *n.s.*, not significative.

*Comparison with all patients without recurrence (n = 971).

°Including adhesiolysis.

**Table 3:** Early post-operative and late outcomes for all patients (n = 1018), recurrences* (n = 47) with significative risk factors (p value), and comparison between IH (n = 665) and PH (n = 353).

	All patients	Recurrence*		Hernia type		
	n = 1018	n = 47	P value	IH (n = 665, 65%)	PH (n = 353, 35%)	P value
All post-operative complications	37 (3.6)	4 (8.5)	0.051	25 (3.8)	10 (2.9)	n.s
**Surgical post-operative complications**						
Abdominal wall hematoma	12 (1.2)	0 (0.0)	n.s	6 (0.9)	6 (1.7)	n.s
Hemoperitoneum	3 (0.3)	0 (0.0)	n.s	2 (0.3)	1 (0.3)	n.s
Peritonitis^	5 (0.5)	2 (4.3)	< 0.001	5 (0.8)	0 (0.0)	n.s
Prosthesis infection^	5 (0.5)	2 (4.3)	< 0.001	5 (0.8)	0 (0.0)	n.s
Bowel obstruction	5 (0.5)	1 (2.1)	n.s	5 (0.8)	0 (0.0)	n.s
Medical complication	13 (1.3)	1 (2.1)	n.s	10 (1.5)	3 (0.9)	n.s
Reintervention^§^	9 (0.9)	3 (6.4)	< 0.001	8 (1.2)	1 (0.3)	n.s
Prosthesis removal	5 (0.5)	2 (4.3)	n.s	5 (0.8)	0 (0.0)	n.s
NRS pain scale (median [Q1–Q3])	2 (0–2)	1 (1–3)	0.022	1 (0–3)	2 (1–2)	n.s
**NRS pain scale**						
None	191 (18.9)	10 (21.3)	n.s	131 (19.9)	60 (17.1)	0.059
Mild	474 (47.0)	24 (51.1)		278 (42.2)	196 (56.0)	
Moderate	76 (7.5)	7 (14.9)		50 (7.6)	26 (7.4)	
Severe	10 (1.0)	0 (0.0)		8 (1.2)	2 (0.6)	
Undocumented pain	267 (26.5)	6 (12.8)		198 (30.0)	69 (19.7)	
Hospital stay (mean days SD, range)	2.9 ± 2.8 (1–44)	4.1 ± 6.4 (1–44)	0.003	3.4 ± 3.0 (1–44)	1.9 ± 1.9 (1–26)	< 0.001
All late surgical-related complications (> 30 days)	133 (13.2)	2 (4.3)	0.066	62 (9.4)	71 (20.3)	< 0.001
**Late surgical-related complications**						
Persistent/symptomatic seroma	111 (11.0)	1 (2.1)	n.s	43 (6.5)	68 (19.4)	< 0.001
Adhesion occlusive bowel syndrome	2 (0.2)	0 (0.0)		2 (0.3)	0 (0.0)	
Mesh infection	1 (0.1)	0 (0.0)		0 (0.0)	1 (0.3)	
Chronic pain	19 (1.9)	1 (2.1)		17 (2.6)	2 (0.6)	
Bulging	34 (3.4)	4 (8.5)	0.044	31 (4.7)	3 (0.9)	0.001
Hernia recurrence	47 (4.7)	–	–	44 (6.7)	3 (0.9)	< 0.001
Follow-up months (median, Q1–Q3)	26.3 (14.0–41.8)	43.0 (26.5–78.1)	< 0.001	26.4 (13.3–44.9)	24.8 (12.3–33.3)	< 0.001
Follow-up months (mean ± SD, range)	30.4 ± 21.4 (12.1–102.7)	50.7 ± 27.5 (12.2–98.3)	< 0.001	32.8 ± 23.3 (12.1–102.7)	25.8 ± 16.2 (12.2–93.7)	< 0.001
Mortality	0 (0.0)	0 (0.0)	–	0 (0.0)	0 (0.0)	–

Data presented were n (%), unless specified. *Q1*; first quartile, *Q3*; third quartiles; *n.s.*, not significative.

*Comparison with all patients without recurrence (n = 971).

°6 patients presented more than one complication.

^All patients with peritonitis presented infection of the mesh.

^§^Reinterventions were managed laparoscopically in 2 cases and with open approach in 7 cases.

Mean patient age was 60.4 ± 13.6 (range 20–92), with an equal distribution of male and female patients (49.7% and 50.3%, respectively) and over one third (35.7%) of obese patients (BMI ≥ 30). Patients presenting with incisional hernia were the 65.3% (n = 665), with 84.2% of primary incisional and 12.8% of recurrent incisional defects; the remaining 34.7% (n = 353) were primary hernias. Most hernias were small (W1; 49.7%) and located medially (89.7%). Border hernias were observed in 4.6% of patients (4.6%), Table 1.

Overall, most interventions were performed electively (95%). Strangulated hernia was observed in 5.7% cases and were treated with emergent repair (Table 2). Of note, no cases of intra-operative bowel resections were registered.

Mesh size was selected according to hernia defect and the median mesh overlap was 5 cm (range 4–11). Absorbable mesh fixation to the abdominal wall was most commonly used (47.2%) (Table 2). In some (n = 53) small hernia defects in non-obese patients, surgeons performed hernia closure mainly for internal training and comparison purposes, and defect closure was performed with a non-absorbable or long-term absorbable monofilament suture.

The overall short-term post-operative complications rate was 3.6%, with 9 cases (0.9%) requiring surgical reinterventions during hospitalization. Overall mean hospital stay was 2.9 ± 2.8 days (range 1–44). No readmissions or patient deaths were registered within 30 days. The mean follow-up was 30.4 months ± 21.4 (12.1–102.7 (Table 3).

### Incisional hernia and primary hernias analysis

Baseline characteristics of the IH and PH patients are outlined in Table 1.

Data confirmed that IH patients are older (< 0.001), more frequently female (p = 0.007), less frequently obese (0.031), classified at higher ASA class surgical risk (p < 0.001), less frequently smokers (p < 0.001) and presented with significantly different hernia characteristics (more frequently larger defects W2-W3, located at lateral and border sites, swiss cheese type and presenting with adhesion syndrome, p < 0.001), compared to PH patients. Adhesiolysis was most frequently required in IH group than PH (11.6% vs 3.4%, p < 0.001). The most common fixation system used for IH was absorbable type (58%). Intraoperative complications were infrequent (1.2%) in IH group: they included 6 cases of enterotomies, either occurred during adhesiolysis or caused by traction maneuvers during the reduction of hernia content and all safely managed laparoscopically during the same intervention with suture repair, and 2 cases of major bleeding secondary to an epigastric artery injury during mesh fixation (treated with transparietal artery ligation). There were 2 conversions to open repair due to massive adhesion syndrome (Table 2). Early post-operative complications amounted to 3.8% and reinterventions to 1.2%. The 8 cases requiring surgical reinterventions included 1 case of abdominal wall bleeding treated laparoscopically with a laivage-drainage procedure, and 7 cases (2 bowel obstructions and 5 perforated peritonitis) managed with open repair. In all cases of contaminated abdomen due to peritonitis for bowel injury (n = 5) the open approach involved mesh removal.

Late surgical complications were registered in < 10% of patients with IH throughout the follow-up of 26 months; cases were associated with persistent/symptomatic seroma in 6.5% and with chronic pain in 2.6%, including one case requiring a reintervention for tack removal. We recorded 2 cases of bowel obstruction due to adhesion syndrome (0.3%), that were treated with laparoscopic adhesiolysis and mesh removal. Bulging presented in 4.7% (Table 3).

PH patients were mostly treated with non absorbable fixation systems (59.4%), adhesiolysis was less frequently required than IH (p < 0.001), and intra-operative complications were < 1%, including 2 enterotomies and 1 major bleeding (treated as outlined above). There was no cases of conversion to open repair reported (Table 2). Less than 3% of patients encountered early post-operative complications and only 1 required a reintervention for hemoperitoneum, managed laparoscopically. Throughout the follow-up of 25 months, 20% were reported to have surgical-related complications, almost 96% of which were persistent/symptomatic seroma. Bulging was infrequently noted (0.9%) (Table 3).

In comparison, operative time and hospital stay were confirmed to be longer for IH than PH (p < 0.001), but intraoperative complications, early post-operative complications and reinterventions were comparable between the groups (Tables 2, 3).

Despite a longer follow-up in IH patients (< 0.001), late surgical related complications were less frequently registered compared to the PH group (mainly due to the higher incidence of late persistent/symptomatic seroma in PH (p < 0.001), see Table 3.

### Recurrence and predictive risk factors

**Figure 1:**
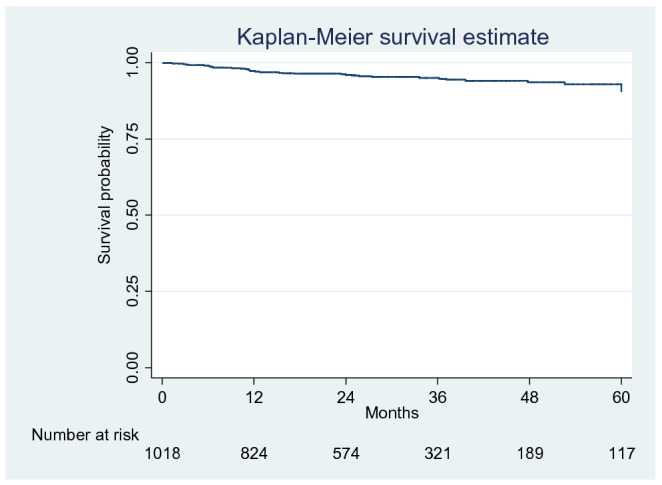
Kaplan–Meier survival curve for time to recurrence for all 1018 patients. Overall recurrence-free survival was 97%, 95% and 88% at 12 months, 36 months and 60 months, respectively.

The overall recurrence rate for the total cohort of patients was 4.7% (n = 47) at a mean follow-up of 30.4 months. Over half (25/47; 53%) of the recurrences were registered within 12 months of LVHR surgical intervention and overall recurrence-free survival was 97%, 95% and 88% at 1, 3 and 5 years respectively (Fig. 1).

**Figure 2:**
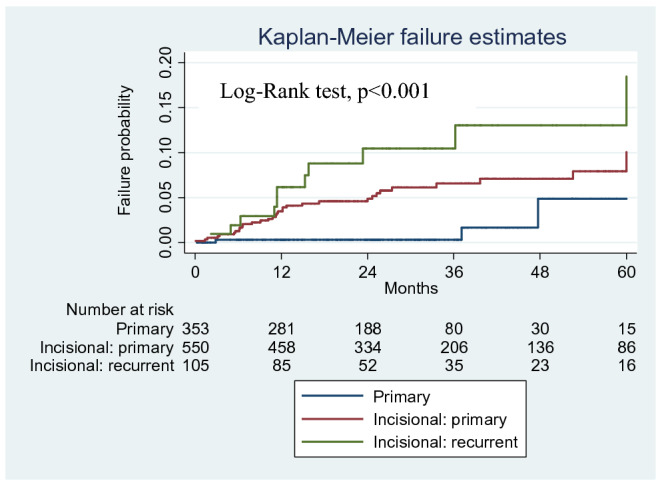
Kaplan–Meier survival curve for hernia recurrence; comparison for type of hernia (PH; primary IH; recurrent IH), Log rank, p < 0.001. Recurrence is defined as failure probability.

IH group presented a higher rate of recurrence than PH (6.7% vs 0.9%, p < 0.001), with almost all cases registered among IH patients (n = 44) (Table 3). In particular, recurrence rates according to hernia type were 0.85% for PH (3/353), 5.9% for primary IH (33/560) and 10.5% for recurrent IH (11/105), p < 0.001, see Fig. 2.

As were insufficient cases of recurrence among PH patients, predictive factors evaluation was performed for the IH population only. Tables 4 and 5 report uni and multi-variate analyses for IH. Interestingly, at univariable analysis on 665 IH cases BMI, hernia size and location were not found to be predictive factors of recurrence, as well being primary incisional or recurrent incisional hernia (Table 4). Post-operative complications presented a tendency to recurrence (p = 0.055), and among cases requiring reintervention (n = 8) recurrence was registered in 3 patients (p = 0.001), all treated with an open surgical approach (secondarily to peritonitis and bowel obstruction in 2 and 1 cases respectively). Multivariate analysis identified the application of absorbable tacks and reintervention as major risk factors, increasing risk of recurrence by 2.94 and 2.89 times, respectively.

**Table 4:** Univariable analysis of independent risk factors and relative risk of IH recurrence.

	IH (n = 665)	Recurrence* (n = 44)	P value
Age (years, median [Q1–Q3])	65 (54–73)	65 (52–73)	n.s
Age years, mean ± SD (range)	63.1 ± 12.6 (28–92)	62.4 ± 13.4 (39–90)	n.s
**Sex**			
Male	310 (46.6)	23 (52.3)	n.s
Female	355 (53.4)	21 (47.7)	
**BMI (mean kg/m**^**2**^**)**			
Normal < 30	421 (63.3)	27 (61.4)	n.s
Obesity ≥ 30	221 (33.2)	17 (38.6)	
BMI mean [± SD]	28.2 ± 5.5 (17–69)	27.7 ± 5.7 (20–43.5)	n.s
**ASA class**			
I	96 (14.4)	7 (15.9)	n.s
II	409 (61.5)	27 (61.4)	
III	156 (23.5)	8 (18.2)	
IV	2 (0.3)	1 (2.3)	
**Clinical comorbidities**			
COPD	31 (4.7)	1 (2.3)	n.s
Cardiac comorbidities	62 (9.3)	3 (6.8)	n.s
Diabetes mellitus	81 (12.2)	3 (6.8)	n.s
Hypertension	290 (43.6)	21 (47.7)	n.s
Current smoker	130 (19.5)	9 (20.4)	n.s
Oral anticoagulant therapy	30 (4.5)	0 (0)	n.s
Steroid therapy	12 (1.8)	0 (0)	n.s
Radiotherapy	9 (1.4)	1 (2.3)	n.s
Chirrosis	6 (0.9)	1 (2.3)	n.s
**Hernia characteristics**			
Primary IH	560 (84.2)	33 (75)	n.s
Recurrent IH	105 (15.8)	11 (25)	
Swiss cheese	169 (25.4)	6 (13.6)	n.s
Adhesion syndrome	77 (11.6)	6 (13.6)	n.s
**Hernia defect size**			
W1 (< 4 cm)	192 (28.9)	13 (29.5)	n.s
W2 (≥ 4 to < 10 cm)	348 (52.3)	22 (50)	
W3 (≥ 10 cm)	125 (18.8)	9 (20.5)	
**Hernia localization**			
Medial	573 (86.2)	35 (79.5)	n.s
Lateral	81 (12.2)	8 (18.2)	
Combined	11 (1.7)	1 (2.3)	
Border	46 (6.9)	5 (11.4)	n.s
**Intervention type**			
Elective	634 (96.2)	44 (100)	n.s
Emergent	30 (4.6)	0 (0)	
**Operative time**			
(Median, IQR)	80 (60–120)	80 (60–122)	n.s
Mean ± SD (range)	89.2 ± 39.7 (19–300)	94.4 ± 44.7 (20–195)	n.s
**Fixation system**			
Absorbable	382 (58.0)	29 (65.9)	n.s
Permanent	177 (26.9)	6 (13.6)	
Mixed	106 (16.1)	9 (20.5)	
Mesh overlap mean cm ± SD (range)	5.1 ± 0.8 (4–11)	5.3 ± 1.0 (4–9)	n.s
Closure of hernia defect	43 (6.5)	5 (11.4)	n.s
All concurrent laparoscopic procedures	99 (15.0)	9 (20.5)	n.s
**Concurrent laparoscopic procedures**			
Adhesiolysis	77 (11.6)	6 (13.6)	n.s
Cholecystectomy	11 (1.7)	1 (2.3)	n.s
Inguinal hernia repair	9 (1.4)	2 (4.5)	0.058
Cholecystectomy + Inguinal hernia repair	2 (0.3)	3 (6.8)	n.s
Ovarian kyst asportation	1 (0.2)	0 (0)	n.s
Adrenalectomy	1 (0.2)	0 (0)	n.s
Appendectomy	0 (0.0)	0 (0)	n.s
**All intraoperative complications**	8 (1.2)	0 (0)	n.s
Bowel injury	6 (0.9)	0 (0)	n.s
Major bleeding	2 (0.3)	0 (0)	n.s
Conversion to open repair	2 (0.3)	0 (0)	n.s
All post-operative complications^^^	25 (3.8)	4 (9.1)	0.055
**Surgical post-operative complications**			
Abdominal wall hematoma	6 (0.9)	0 (0)	n.s
Hemoperitoneum	2 (0.3)	0 (0)	n.s
Peritonitis^^^^	5 (0.8)	2 (4.5)	0.003
Prosthesis infection^^^^	5 (0.8)	2 (4.5)	0.003
Bowel obstruction	5 (0.8)	1 (2.3)	n.s
Medical complication	10 (1.5)	1 (2.3)	n.s
Reintervention^§^	8 (1.2)	3^§§^ (6.8)	0.001
Prosthesis removal	5 (0.8)	2 (4.5)	0.003
NRS pain scale (median [Q1–Q3])	1 (0–3)	1 (1–3)	n.s
**NRS pain scale**			
None	131 (19.9)	8 (18.2)	n.s
Mild	278 (42.2)	23 (52.3)	
Moderate	50 (7.6)	7 (15.9)	
Severe	8 (1.2)	0 (0)	
Undocumented pain	198 (30.0)	6 (13.6)	
Hospital stay (mean days SD, range)	3.4 ± 3.0 (1–44)	4.1 ± 6.4 (1–44)	0.003
All late surgical-related complications (> 30 days)	62 (9.4)	2 (4.5)	n.s
**Late surgical-related complications**			
Persistent/symptomatic seroma	43 (6.5)	1 (2.3)	n.s
Bowel occlusion	2 (0.3)	0 (0)	
Mesh infection	0 (0.0)	0 (0)	
Chronic pain	17 (2.6)	1 (2.3)	
Bulging	31 (4.7)	4 (9.1)	n.s
Follow-up months (median, Q1–Q3)	26.4 (13.3–44.9)	45.5 (26.5–78.1)	0.001
Follow-up months mean ± SD (range)	32.8 ± 23.3 (0.1–102.7)	51.0 ± 27.8 (2.2–98.3)	< 0.001
Mortality	0 (0.0)	0 (0)	–

Patient baseline demographic, clinical and hernia characteristics, intraoperative, early post-operative and long-term data for IH subgroup with Kaplan–Meier survival analysis and identification of risk factors for recurrence. Number of patients (n = 665), number of recurrences* (n = 44) and p value.

Data presented were n (%), unless specified. *Q1*; first quartile, *Q3*; third quartiles; *BMI*, body mass index; *ASA*, American Society of Anesthesiologists; *COPD*, chronic obstructive pulmonary disease; *n.s.*, not significative.

*Comparison with all patients without recurrence (n = 621).

°Including adhesiolys; ^^^6 patients presented more than one complication; ^^^^All patients with peritonitis presented infection of the mesh; ^§^Reinterventions were managed laparoscopically in 1 case and with open approach in 7 cases; ^§§^ reintervention was laparotomic in all 3 cases.

**Table 5:** Multivariable analysis of independent risk factors and relative risk of IH recurrence; HR (Hazard Ratio), 95% CI (Confidence Interval) and p value.

Risk factor	HR	95% CI	p value
**BMI (mean kg/m**^**2**^**)**			
Normal < 30	ref		
Obesity ≥ 30	1.10	(0.59–2.04)	0.742
**Hernia characteristics**			
Primary	ref		
Recurrent incisional	1.81	(0.90–3.63)	0.093
**Hernia localization**			
Medial	ref		
Lateral	1.91	(0.87–4.15)	0.102
Combined	1.32	(0.16–10.37)	0.788
Border	2.23	(0.83–5.95)	0.108
**Fixation system**			
Permanent	ref		
Absorbable	2.94	(1.18–7.31)	0.020
Mixed	2.54	(0.88–7.32)	0.083
Reintervention	2.89	(1.59–5.27)	< 0.001

Surgical management of recurrent hernia was adopted in 12 cases (all IH patients); 7 cases of laparoscopic approach with additional mesh placed overlapping the original mesh, and 5 cases of conventional open approach with mesh removal and application of a different type of mesh.

### Fixation system sub-analysis

The choice of the fixation systems was analyzed according to all patients and hernia characteristics. As reported in Table 6, data shows that the absorbable fixation tacks were more frequently selected for slightly elderly patients (p value > critical value), diabetic patients (p = 0.043), W2 and W3 hernias (p < 0.001) and, as is evident in Table 2, IH patients (p < 0.001). Interestingly, the type of fixation was not influenced by other patient characteristics, including BMI.

**Table 6:** Patient baseline demographic, clinical and hernia sizes according to the type of fixation system.

		Type of fixation system			P value
		Not absorbable (n = 385)	Absorbable (n = 480)	Mixed (n = 153)	
Sex	Male (n = 506)	202 (39.9)	234 (46.2)	70 (13.9)	0.316
	Female (n = 512)	183 (35.8)	246 (48)	83 (16.2)	
Age (years, median)		58.8	61.6		4.2067*
			61.6	61.07	0.5935*
		58.8		61.07	2.4451*
BMI (mean kg/m^2^)	≥ 30 (n = 363)	149 (41.04)	151 (41.6)	63 (17.3)	0.146
**Clinical comorbidities**					
COPD	(n = 47)	10 (21.3)	28 (59.5)	9 (19.2)	0.056
Diabetes mellitus	(n = 119)	33 (27.7)	63 (52.9)	23 (19.4)	0.043
Hypertension	(n = 430)	153 (35.5)	213 (49.5)	64 (14.9)	0.374
Oral anticoagulant	(n = 44)	11 (25)	25 (56.8)	8 (18.2)	0.197
Steroid therapy	(n = 14)	5 (35.7)	7 (50)	2 (14.3)	0.976
Radiotherapy	(n = 11)	4 (36.3)	7 (63.7)	0	0.313
Current smoker	(n = 246)	126 (51.3)	83 (33.7)	37 (15)	< 0.001
Chirrosis	(n = 7)	3 (42.8)	3 (42.8)	1 (14.4)	0.962
Hernia size defect	W1 (< 4 cm) (n = 506)	257 (50.8)	178 (35.1)	71 (14.1)	< 0.001
	W2 (≥ 4 to < 10 cm) (n = 384)	92 (24)	225 (58.6)	67 (17.4)	
	W3 (≥ 10 cm) (n = 128)	36 (28.1)	77 (60.1)	15 (11.8)	

Data presented were n (%), unless specified. *BMI*, body mass index; *COPD*, chronic obstructive pulmonary disease.

*Tukey–Kramer pairwise comparisons for variable fixation studentized range critical value (.05, 3, 1007) = 3.3194419.

## Discussion

The first LVHR IPOM was presented by Le Blanc et al. in 1992^7^ and since then it has progressively been considered a valid alternative to open ventral hernia repair^1,8^, offering lower morbidity rates^8,10^, less SSI rates^8–10,12^ and shorter hospital stay^8–11^, with similar recurrence incidence^6,8,12^. Nowadays, most LVHR series include heterogeneous patient cohorts with different hernia characteristics undergoing different surgical approaches^3,13^. Moreover, PH and IH are pooled together, despite evidence that they are two distinct pathological entities^4^. Laparoscopic repair has been reported to have similar^1,5,8,21–23^, if not lower^8,24^, recurrence rates compared with open repair, but as described by Köckerling et al.^15^, in most series comparative data include pooled PH and IH results, varied surgical approaches and mesh applications, with the risk of erroneous interpretations of surgical effectiveness. Currently, there are few meta-analyses comparing laparoscopic and open techniques for IH repair^23,25^, and most data available are limited by short term follow up^4,25^.

In our series, a single type of light-weight polypropylene mesh with hydrogel barrier was employed for all patients. This mesh has reported acceptable rates of post-operative complications with standard laparoscopic repair^26,27^. Several randomized studies and meta-analysis have demonstrated that most barrier composite meshes present low risk when placed intraperitoneally^5,8,26^. Some papers have also compared different prosthetic materials^28^ but short and long-term outcomes of different types of mesh are still lacking^8^.

Other technical features of the LVHR procedure (including mesh fixation device) are hypothesized to influence surgically related complications and recurrences^17^. The IEHS guidelines^29^ and the most recent Italian Consensus^17^ have developed specific criteria of application, indications and contraindications for the LVHR technique according to patients’ clinical and surgical settings. However, there are still questions regarding predictive factors for recurrence after LVHR^2,8,25^, and the efficacy of LVHR, particularly for IH, and the associated risk factors for the success of this technique are still debated. The present study reports one of the largest series of a standardized LVHR approach, with the application of a single mesh type, and an assessment of barriers to technical success.

In this series, differences between IH and PH subgroups were clearly highlighted, and as expected, surgical repair of IH resulted more complex than PH, mostly due to older age (< 0.001), higher ASA class (p < 0.001), more complex defect to manage (larger, more frequently at border sites, swiss cheese type, p < 0.001), presenting more frequently with adhesion syndrome (p < 0.001) and requiring longer operative time (p = 0.032). All these variables, within a complex setting, can increase the risk of intra-operative bowel injury, missed enterotomies and subsequent post-operative morbidity, as affirmed also by ten Broek et al.^30^. Previous authors have suggested that postoperative peritonitis after laparoscopic procedures are rare, but missed intraoperative enterotomies lead to serious complications, and represent a major adverse surgical outcome of LVHR compared to open approach, with incidences of up to 3.7%^8,31^ and a mortality rate of 7.7%^32^. The current study registered 5 cases (0.7%) of post-operative peritonitis due to bowel injury with concurrent mesh infection in IH population, while no cases resulted in PH group. However, despite IH can be surgically challenging, this study did not report a significative difference in intra- or post-operative complications or reinterventions for IH compared to PH, and given the overall improved outcomes compared to those available in literature, the laparoscopic procedure seems to be safe and effective for all types of abdominal hernia repair.

IH confirmed to be more exposed to develop recurrence than PH (p < 0.001). The overall recurrence rate for IH reported of 6.7%, over a mean follow up of 32.8 ± 23.3 months, is within the lower portion of the range of recurrence published in literature for LVHR, between 3 and 15%^2,8,23,32^. Recently, Mercoli et al. in a prospective series of 417 patients treated with LVHR IPOM, presented higher recurrence rates among hernia types: PH (range 4.6–10% according to complication), primary IH (14.5%) and recurrent IH (10–40% according to complication)^32^, but we know that different incidences of recurrence may be associated with study design (prospective vs retrospective), with any aspect of the technique and the selection of mesh and with the completeness and precision of the follow-up strategy of recurrence assessment. Clear individuation and comparisons of risk factors for surgical outcomes are difficult to make as the discrepancies may be due to the relative ratios of PH/IH in cohorts with combined hernia types. In this case, all the aforementioned variables were supposed to be identified as risk factors for recurrence in IH patients. Interestingly, BMI, hernia size, border hernias and swiss cheese type were not predictive factors, whilst the use of absorbable tacks and reoperation due to post-operative complications were associated with recurrence as revealed by multivariate statistics. Given the limited cases of recurrence among PH patients, specific risk factors could only be estimated for IH ones.

In this series we found a tendency to apply absorbable tacks in elderly patients, diabetics, W2 and W3 hernias and incisional hernias. Among the IH patients, absorbable tacks were predominantly used (58%), comparing to non absorbable (26.9%) and mixed (16.1%) systems. The use of absorbable fixation systems resulted in increasing the likelihood of recurrence by almost 3 times (p = 0.020). The only aspect of the LVHR surgical approach presented in this current series that was not standardized was the surgeons’ choice of fixation system, still a highly debated aspect of LVHR procedure ^17,33–35^. Christoffersen et al. described that recent exhaustive attempts to diagnose recurrence have not identified other risk factors, apart from fixation devices^36^. Authors suggest that the most critical period for recurrence is the first year following mesh implantation, as the prosthesis takes 12 months to fully integrate into the host tissue; accordingly, many recurrences observed with absorbable fixation systems were registered within the first 12 months, with a higher incidence in comparison with mixed and non-absorbable devices. The time to reabsorption for absorbable tacks may be insufficient to enable the mesh to integrate into the host tissue, and when used alone they are at higher risk than permanent fixation systems to ensure sufficient tensile strength in terms of resistance. The authors hypothesize that for a mixed fixation system (comprised of a 1:1 ratio of absorbable and non-absorbable tacks), a 50% long term material of fixation seems to decrease the risk of recurrence, ensuring higher mechanical resistance and suggesting that the number of tacks used may also influence recurrence risk. Therefore, the authors recommend the application of non absorbable tacks or at least a mixed fixation system, but further studies are needed to investigate the safety and efficacy of this hypothesis.

Type of mesh fixation has also been associated with postoperative pain^17,34^. We know that the right balance between the correct fixation to prevent recurrence and excessive fixation, likely causing post-operative pain is mandatory for surgeons^34,35^. In their meta-analysis Khan et al. reported a similar incidence of recurrence and chronic pain for absorbable and permanent tacks^34^, whilst similarly to our study, Christoffersen et al. in 2015^36^ identified absorbable tacks as an independent risk factor for recurrence, with rates of moderate or severe chronic pain associated to the types of tacks utilized. The current data in terms of recurrences according to the type of fixation system used, supported by the overall low chronic pain registration among treated patients in this series, suggest that:

- Absorbable fixation devices should not be used alone
- LVHR outcomes are improved with the use of non-absorbable fixation system
- Mixed fixation systems should be reserved for cases of reducing the risk of chronic pain.

Further studies dedicated to the quantification of the percentage of absorbable tacks which can be safely used is greatly needed.

Recently, new minimally invasive techniques have been introduced to overcome limitations of laparoscopic and open ventral hernia repair, including laparoscopic, endoscopic, robot-assisted and hybrid procedures, intended to identify alternative methods of placing the mesh in the sublay retromuscular position, and to combine extraperitoneal techniques with a posterior component separation or transversus abdominis release procedures, when required. For example, Reinpold et al.’s MILOS or EMILOS techniques^37^, Daes et al.’s eTEP approach^38^ and other early outcomes seem promising^39^. Further studies are required to identify and validate the most appropriate technical management approach according to patient characteristics and abdominal wall hernia defect type.

The current study is limited by the retrospective methodological design which may be biased in patient selection and complete data retrieval. Some aspects, such as patient comorbidities, may be underestimated. As mentioned, a final attempt to contact all patients was made at study closure by phone but not all patients were clinically visited. Some data were not analyzed, such as the association of pain and fixation systems, as not all included patients’ post-operative pain was recorded. Due to small numbers, the distinction between reintervention by laparoscopic or open approaches could not be performed; it should however be noted that all cases of reintervention-related hernia recurrence were associated with open repair and subsequent compromised mesh integrity. Data regarding precise mesh size was not collected (only mesh overlap) but in light of new guidelines, future studies should include mesh area-to-defect area ratio and analyze whether this can effect recurrence^40^. These data should be investigated further in future studies.

## Conclusions

The current study suggests that LVHR procedure with a light-weight polypropylene mesh has low intra-operative complication rates and acceptable post-operative and late complication rates for both IH and PH. IH patients are at a much higher risk of recurrence and surgical strategy should prefer non absorbable or mixed fixation systems. The current authors suggest that absorbable tacks alone should be avoided in LVHR interventions.

All data generated or analyzed during the current study are available from the corresponding author on reasonable request.
